# Prevalence and Factors Associated with Drooling in Parkinson's Disease: Results from a Longitudinal Prospective Cohort and Comparison with a Control Group

**DOI:** 10.1155/2023/3104425

**Published:** 2023-04-06

**Authors:** Diego Santos-García, Teresa de Deus Fonticoba, Carlos Cores Bartolomé, Maria J. Feal Painceiras, Maria Cristina Íñiguez-Alvarado, Silvia Jesús, Maria Teresa Buongiorno, Lluís Planellas, Marina Cosgaya, Juan García Caldentey, Nuria Caballol, Ines Legarda, Jorge Hernández Vara, Iria Cabo, Lydia López Manzanares, Isabel González Aramburu, Maria A. Ávila Rivera, Víctor Gómez Mayordomo, Víctor Nogueira, Víctor Puente, Julio Dotor García-Soto, Carmen Borrué, Berta Solano Vila, María Álvarez Sauco, Lydia Vela, Sonia Escalante, Esther Cubo, Francisco Carrillo Padilla, Juan C. Martínez Castrillo, Pilar Sánchez Alonso, Maria G. Alonso Losada, Nuria López Ariztegui, Itziar Gastón, Jaime Kulisevsky, Marta Blázquez Estrada, Manuel Seijo, Javier Rúiz Martínez, Caridad Valero, Mónica Kurtis, Oriol de Fábregues, Jessica González Ardura, Ruben Alonso Redondo, Carlos Ordás, Luis M. L. López Díaz, Darrian McAfee, Pablo Martinez-Martin, Pablo Mir, Study Group COPPADIS

**Affiliations:** ^1^CHUAC,Complejo Hospitalario Universitario de A Coruña, A Coruña, Spain; ^2^CHUF,Complejo Hospitalario Universitario de Ferrol, A Coruña, Spain; ^3^Unidad de Trastornos del Movimiento, Servicio de Neurología y Neurofisiología Clínica, Instituto de Biomedicina de Sevilla, Hospital Universitario Virgen del Rocío, CSIC, Universidad de Sevilla, Seville, Spain; ^4^CIBERNED (Centro de Investigación Biomédica en Red Enfermedades Neurodegenerativas), Madrid, Spain; ^5^Hospital Universitari Mutua de Terrassa, Terrassa, Barcelona, Spain; ^6^Clínica del Pilar, Barcelona, Spain; ^7^Hospital Clínic de Barcelona, Barcelona, Spain; ^8^Centro Neurológico Oms 42, Palma de Mallorca, Spain; ^9^Consorci Sanitari Integral, Hospital Moisés Broggi, Sant Joan Despí, Barcelona, Spain; ^10^Hospital Universitario Son Espases, Palma de Mallorca, Spain; ^11^Hospital Universitario Vall d'Hebron, Barcelona, Spain; ^12^Complejo Hospitalario Universitario de Pontevedra (CHOP), Pontevedra, Spain; ^13^Hospital Universitario La Princesa, Madrid, Spain; ^14^Hospital Universitario Marqués de Valdecilla, Santander, Spain; ^15^Consorci Sanitari Integral, Hospital General de L´Hospitalet, L´Hospitalet de Llobregat, Barcelona, Spain; ^16^Hospital Universitario Clínico San Carlos, Madrid, Spain; ^17^Hospital Da Costa, Burela, Lugo, Spain; ^18^Hospital del Mar, Barcelona, Spain; ^19^Hospital Universitario Virgen Macarena, Sevilla, Spain; ^20^Hospital Infanta Sofía, Madrid, Spain; ^21^Institut d'Assistència Sanitària (IAS), Institut Català de La Salut, Girona, Spain; ^22^Hospital General Universitario de Elche, Elche, Spain; ^23^Fundación Hospital de Alcorcón, Madrid, Spain; ^24^Hospital de Tortosa Verge de La Cinta (HTVC), Tortosa, Tarragona, Spain; ^25^Complejo Asistencial Universitario de Burgos, Burgos, Spain; ^26^Hospital Universitario de Canarias, San Cristóbal de La Laguna, Santa Cruz de Tenerife, Spain; ^27^Hospital Universitario Ramón y Cajal, IRYCIS, Madrid, Spain; ^28^Hospital Universitario Puerta de Hierro, Madrid, Spain; ^29^Hospital Álvaro Cunqueiro, Complejo Hospitalario Universitario de Vigo (CHUVI), Vigo, Spain; ^30^Complejo Hospitalario de Toledo, Toledo, Spain; ^31^Complejo Hospitalario de Navarra, Pamplona, Spain; ^32^Hospital de Sant Pau, Barcelona, Spain; ^33^Hospital Universitario Central de Asturias, Oviedo, Spain; ^34^Hospital Universitario Donostia, San Sebastián, Spain; ^35^Hospital Arnau de Vilanova, Valencia, Spain; ^36^Hospital Ruber Internacional, Madrid, Spain; ^37^Hospital de Cabueñes, Gijón, Spain; ^38^Universitario Lucus Augusti (HULA), Lugo, Spain; ^39^Hospital Rey Juan Carlos, Madrid, Spain; ^40^Complejo Hospitalario Universitario de Orense (CHUO), Orense, Spain; ^41^University of Maryland School of Medicine, Baltimore, MD, USA; ^42^Fundación Degen, C/Juana de Vega 23 2°, A Coruña 15004, Spain

## Abstract

**Introduction:**

Drooling in Parkinson's disease (PD) is frequent but often goes underrecognized. Our aim was to examine the prevalence of drooling in a PD cohort and compare it with a control group. Specifically, we identified factors associated with drooling and conducted subanalyses in a subgroup of very early PD patients. *Patients and Methods*. PD patients who were recruited from January 2016 to November 2017 (baseline visit; V0) and evaluated again at a 2-year ± 30-day follow-up (V2) from 35 centers in Spain from the COPPADIS cohort were included in this longitudinal prospective study. Subjects were classified as with or without drooling according to item 19 of the NMSS (Nonmotor Symptoms Scale) at V0, V1 (1-year ± 15 days), and V2 for patients and at V0 and V2 for controls.

**Results:**

The frequency of drooling in PD patients was 40.1% (277/691) at V0 (2.4% (5/201) in controls; *p* < 0.0001), 43.7% (264/604) at V1, and 48.2% (242/502) at V2 (3.2% (4/124) in controls; *p* < 0.0001), with a period prevalence of 63.6% (306/481). Being older (OR = 1.032; *p* = 0.012), being male (OR = 2.333; *p* < 0.0001), having greater nonmotor symptom (NMS) burden at the baseline (NMSS total score at V0; OR = 1.020; *p* < 0.0001), and having a greater increase in the NMS burden from V0 to V2 (change in the NMSS total score from V0 to V2; OR = 1.012; *p* < 0.0001) were identified as independent predictors of drooling after the 2-year follow-up. Similar results were observed in the group of patients with ≤2 years since symptom onset, with a cumulative prevalence of 64.6% and a higher score on the UPDRS-III at V0 (OR = 1.121; *p* = 0.007) as a predictor of drooling at V2.

**Conclusion:**

Drooling is frequent in PD patients even at the initial onset of the disease and is associated with a greater motor severity and NMS burden.

## 1. Introduction

Sialorrhea, commonly referred to as drooling, is defined as excessive saliva beyond the margin of the lip. Drooling can be a complication of Parkinson's disease (PD) and is one of the most prevalent complaints of patients, but it is often underrecognized and undertreated [1]. A wide prevalence range has been reported in the literature, ranging from 10 to 84%, with no significant variation across ethnic groups [2–15]. However, when studies compared PD patients with controls, drooling only occurred in 6–15% of people without PD [5, 6, 15, 16]. The broad range in PD patients is likely due to the lack of a standard definition of and diagnostic criteria for sialorrhea and the differences in the PD population studied and the methods used. Despite these obstacles, drooling has still been found to negatively impact the quality of life (QoL) of both patients and caregivers [5, 12, 13, 17–19]. Sialorrhea may bring repercussions for the psychosocial health of the person who drools and added burden for the caregiver as well (e.g., odor, stained clothes, constant wiping, restricted social life, etc.) [1]. Moreover, drooling is associated with an increased risk of dry mouth, impact on bolus formation, loss of antibacterial effects of saliva, perioral dermatological changes, ulceration, tooth decay, gingivitis, dehydration, candidiasis, halitosis, and increased speech difficulties [20–22]. Drooling in PD patients appears to be primarily related to reduced swallowing efficiency and not to an increase in saliva production [20, 23], as dysphagia is the strongest factor associated with drooling [7, 12, 23]. Other reported factors associated with drooling are orofacial rigidity/hypomimia, lingual bradykinesia, aging, male gender, cognitive impairment, hallucinations, nontremor dominant PD phenotype, longer disease duration, and more advanced disease stage [3–8, 11–14, 23–26].

Although many studies have analyzed the frequency of drooling in PD, there is less information about its prevalence and associated factors in early PD patients and how it impacts QoL and change over time. Some studies have reported a prevalence of about 20% in de novo and untreated PD patients and that prevalence increases in the long term [27, 28]. Our hypothesis was that the prevalence of drooling in early PD patients would be high and would negatively impact QoL. The aim of the current study was to examine the prevalence of drooling, and its progression, in a PD cohort and assess its impact on QoL. Furthermore, we compared the frequency of drooling in PD patients with a control group and analyzed all these aspects in a subgroup of patients from the cohort with a short disease duration of ≤2 years since the onset of the symptoms. Moreover, we identified in both groups, the entire cohort and the subgroup with early PD, factors associated with not only drooling but also drooling severity as well.

## 2. Materials and Methods

PD patients who were recruited from January 2016 to November 2017 (baseline visit; V0) and evaluated again at a 2-year ± 30-day follow-up (V2) from 35 centers in Spain from the COPPADIS cohort [29] were included in this study. The methodology of the COPPADIS-2015 study can be consulted in https://bmcneurol.biomedcentral.com/articles/10.1186/s12883-016-0548-9 [30]. This is a multicenter, observational, longitudinal prospective, and 5-year follow-up study designed to analyze disease progression in a Spanish population of PD patients. All patients included were diagnosed according to the UK PD Brain Bank criteria [31].

Information on sociodemographic aspects, factors related to PD, comorbidity, and treatment were collected. Motor status, nonmotor symptoms (NMS), QoL, and disability were assessed at V0 and at V2 using different validated scales: Hoehn and Yahr (H&Y), UPDRS-III and UPDRS-IV, Freezing of Gait Questionnaire (FOGQ)), Parkinson's Disease Cognitive Rating Scale (PD-CRS), Nonmotor Symptoms Scale (NMSS), Beck Depression Inventory-II (BDI-II), Parkinson's disease sleep scale (PDSS), Neuropsychiatric Inventory (NPI), Questionnaire for impulsive-compulsive disorders in Parkinson's Disease-Rating Scale (QUIP-RS), visual analog scale-pain (VAS-Pain), Visual Analog Fatigue Scale (VAFS)), the 39-item Parkinson's Disease Questionnaire (PDQ-39), the EUROHIS-QOL 8-item index (EUROHIS-QOL8), and ADLS (Schwab and England Activities of Daily Living Scale) [30]. In patients with motor fluctuations, the motor assessment was made during the OFF state (without medication in the last 12 hours) and during the ON state. The assessment was only performed without medication in patients without motor fluctuations. The same evaluation as for the patients, except for the motor assessment, was performed in control subjects at V0 and at V2 (2 years ± 1 month). Furthermore, motor (H&Y, UPDRS-III, and UPDRS-IV) and nonmotor assessment (NMSS and ADLS) was conducted in PD patients at 1 year ± 1 month (V1) [30]. LEED was calculated based on the literature [32].

Subjects were classified as with or without drooling according to item 19 of the NMSS [33]. This item is one of the 30 items on this scale and is included in domain 6 (gastrointestinal tract). This question asks about drooling: “Does the patient dribble saliva during the day?.” The score range is from 0 (without the symptom) to 12 (the most frequent and severe). Subjects with an NMSS-item 19 score = 0 were considered “without drooling,” whereas subjects with an NMSS-item 19 score ≥1 (from 1 to 12) were considered “with drooling.” Drooling was identified at V0, V1, and V2 in patients and at V0 and V2 in controls. The drooling burden was also calculated for PD patients. The score at V0, V1, and V2 and the sum of the score from the three visits (NMSS-Drooling_V0+V1+V2_, from 0 to 36) were calculated. Patients reporting drooling during the three visits were defined as patients with “persistent drooling.” The same method was used to define dysphagia (item 20 of the NMSS) [34] and hypomimia (item 19 of the UPDRS-III during the OFF state) [35].

### 2.1. Statistical Analysis

Data were processed using SPSS 20.0 for Windows. For comparisons between PD patients in the control group and PD patients with and without drooling, the Student's *t*-test, Mann–Whitney *U* test, chi-square test, or Fisher test were used as appropriate (distribution for variables was verified by one-sample Kolmogorov–Smirnov test).

Binary and linear regression models were used for determining independent factors associated with drooling (drooling as the dependent variable) and drooling severity (NMSS-Drooling_V0+V1+V2_ score as the dependent variable), respectively. Variables with univariate associations with *p* values <0.20 were included in a multivariable model, and a backward selection process was used to remove variables individually until all remaining variables were significant at the 0.10 level. For exploring the association between drooling and QoL, linear regression models were used with PDQ-39SI (health-related QoL) and EUROHIS-QOL8 (global QoL) as dependent variables. The total domain scores of the PDQ-39 were expressed as a percentage of the corresponding maximum possible score, and a summary index was obtained as an average of the domain scores (PDQ-39SI). The effect was controlled by age, gender, disease duration, LEDD, comorbidities (total number of non-anti-Parkinsonian drugs [36]), motor (H&Y, UPDRS-III, UPDRS-IV, and FOGQ) and nonmotor (NMSS) status, cognitive function (PC-CRS total score), dysphagia, hypomimia, and autonomy for ADL (ADLS), which were included as covariates in the model [36].

For PD patients, analyses were conducted in the entire cohort and in the subgroup of patients with ≤2 years of disease duration since symptoms' onset (PD ≤ 2 y) at the baseline. The *p* value was considered significant for all analyses when it was <0.05.

### 2.2. Standard Protocol Approvals, Registrations, and Patient Consents

For this study, we received approval from the Comité de Ética de la Investigación Clínica de Galicia in Spain (2014/534; 02/DEC/2014). Written informed consents from all participants in this study were obtained. COPPADIS-2015 was classified by the AEMPS (Agencia Española del Medicamento y Productos Sanitarios) as a postauthorization prospective follow-up study with the code COH-PAK-2014-01.

## 3. Results

**Figure 1:**
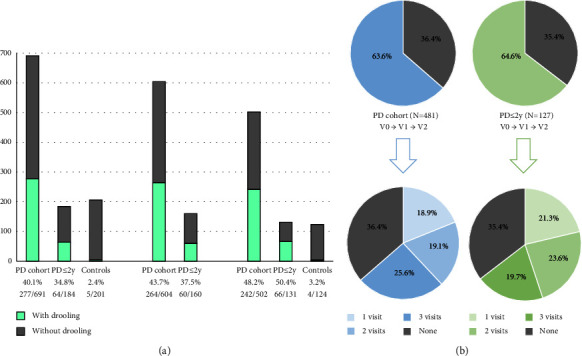
(a) Percentage of patients (the whole cohort and the group with no more than 2 years since symptom onset (PD ≤ 2 y) and controls reporting drooling at different visits: V0, V1, and V2. (b) Prevalence of drooling during the follow-up period in all patients and in the PD ≤ 2 y group who completed the three visits (V1, V2, and V3) and percentage of cases presenting drooling in only 1 visit, 2 visits, and all visits. PD cohort vs. controls at V0, *p* < 0.0001; PD cohort vs. controls at V2, *p* < 0.0001; PD ≤ 2 y group vs. controls at V0, *p* < 0.0001; PD ≤ 2 y group vs. controls at V2, *p* < 0.0001. PD: Parkinson's disease. PD ≤ 2 y group: patients with ≤2 years since symptom onset.

At the baseline, 691 PD patients (62.59 ± 8.92 years old; 60.2% males; mean disease duration 5.5 ± 4.37 years) and 206 patients in the control group (60.98 ± 8.34 years old; 50% males) were considered valid for the analysis. The frequency of drooling in PD patients was 40.1% (277/691) at V0; 43.7% (264/604) at V1; 48.2% (242/502) at V2 (Figure 1(a)). At V0 and V2, drooling was significantly less frequent (*p* < 0.0001) in the control group than in PD patients (2.4% at V0 and 3.2% at V2 in controls). In the patients (*N* = 481; 62.62 ± 8.54 years old, from 35 to 75; 59.2% males) with assessments carried out during all visits (V0, V1, and V2), 63.6% (306/481) of them reported drooling at least once during the study (period prevalence). Specifically, 18.9% (91/481) in only one visit, 19.1% (92/481) in two out of the three visits, and 25.6% (123/481) in all three visits (i.e., persistent drooling) (Figure 1(b)). In the PD ≤ 2 y group (62.22 ± 8.33 years old; 57.3% males; mean disease duration 1.29 ± 0.37 years), the frequency of drooling was 34.8% (64/184) at V0, 37.5% at V1 (60/160), and 50.4% (66/131) at V2. After the 2-year follow-up, the cumulative prevalence of drooling in this group was 64.6% (21.3% in 1 visit, 23.6% in 2 visits, and 19.7% in all visits) (Figure 1(b)).

**Figure 2:**
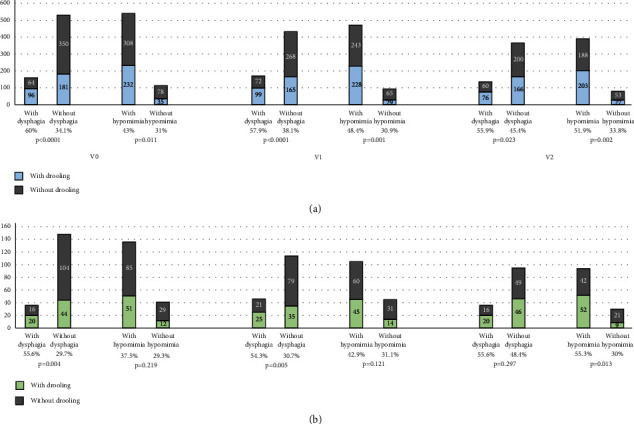
(a) Number of patients reporting drooling at V0, V1, and V2 when they were divided in patients with vs. without dysphagia and with vs. without hypomimia (the whole cohort). A comparison between the percentage is shown for each analysis. (b) Number of patients from the PD ≤ 2 y group reporting drooling at V0, V1, and V2 when they were divided in patients with vs. without dysphagia and with vs. without hypomimia. A comparison between the percentage is shown for each analysis. PD: Parkinson's disease. PD ≤ 2 y group: patients with ≤2 years since symptom onset.

Regarding drooling burden in PD patients, as expected, the NMSS-Drooling_V0+V1+V2_ score was higher in patients with persistent drooling (*p* < 0.0001): drooling in one visit, 1.95 ± 1.67 (*N* = 91); drooling in two out of the three visits, 4.22 ± 2.95 (*N* = 92); persistent drooling, 10.36 ± 6.12 (*N* = 123). Drooling was more frequent in patients with dysphagia than in those without dysphagia: 60% (96/160) vs. 34.1% (181/531) (*p* < 0.0001) at V0; 57.9% (99/171) vs. 38.1% (165/433) (*p* < 0.0001) at V1; 55.9% (76/136) vs. 45.4% (60/166/366) (*p* = 0.023) at V2 (Figure 2(a)). Drooling burden (NMSS-item 19 total score) correlated with dysphagia burden (NMSS-item 20 total score) at V0 (*N* = 691; *r* = 0.322; *p* < 0.0001), at V1 (*N* = 604; *r* = 0.344; *p* < 0.0001), at V2 (*N* = 502; *r* = 0.198; *p* < 0.0001), and after considering all visits together (*N* = 481; *r* = 0.292; *p* < 0.0001). Drooling was also more frequent in patients with hypomimia than in those without hypomimia at V0 (43% vs. 31%; *p* = 0.011), at V1 (48.4% vs. 30.9%; *p* = 0.001), and at V2 (51.9% vs. 33.8%; *p* = 0.002) (Figure 2(a)). A significant correlation was observed between drooling burden and hypomimia burden at V0 (*r* = 0.197; *p* < 0.0001), at V1 (*r* = 0.149; *p* < 0.0001), at V2 (*r* = 0.189; *p* < 0.0001), and after considering all visits (*r* = 0.213; *p* < 0.0001). Similar results were observed in the PD ≤ 2 y group, with significant correlations between drooling burden and dysphagia burden (*r* = 565; *p* < 0.0001) and between drooling burden and hypomimia burden (*r* = 0.360; *p* < 0.00001) after considering the sum of the burden of all visits during the follow-up. Drooling was more frequent in patients with dysphagia at V0 and at V1 and with hypomimia at V2 than in those patients with these symptoms in the PD ≤ 2 y group (Figure 2(b)). Regarding the treatment, none of the patients were receiving botulinum toxin at any of the 3 visits (V0, V1, and V2).

**Figure 3:**
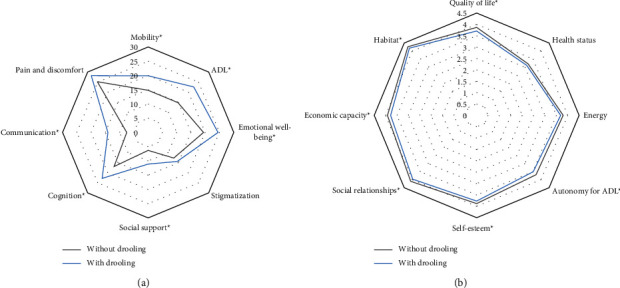
(a) Mean score on each domain of the PDQ-39 at the baseline in PD patients from the entire cohort with vs. without drooling; *p* < 0.0001 for all analysis except for “emotional well-being” (*p* = 0.001), “stigmatization” (*p* = 0.129), and “pain and discomfort” (*p* = 0.063). (b) Mean score on each domain of the EUROHIS-QOL8 at the baseline in PD patients from the entire cohort with vs. without drooling; “quality of life,” *p* = 0.005; “health status,” *p* = 0.178; “energy,” *p* = 0.183; “autonomy for ADL,” *p* = 0.011; “self-esteem,” *p* = 0.033; “social relationships,” *p* = 0.032; “economic capacity,” *p* = 0.020; “habitat,” *p* = 0.046. EUROHIS-QOL8, EUROHIS-QOL 8-item index; PD, Parkinson's disease; PDQ-39, 39-item Parkinson's disease quality of life questionnaire.

At the baseline, drooling was associated with gender (males, 69% vs. 54.3%; *p* < 0.0001), older age (63.79 ± 8.21 vs. 61.8 ± 9.29; *p* = 0.008), and a higher LEDD (646.01 ± 410.21 vs. 512.73 ± 409.22; *p* < 0.0001) (Table 1). Patients with drooling were worse in terms of motor (UPDRS-III; UPDRS-IV; FOGQ) and nonmotor (PD-CRS; NMSS; BDI-II; NPI; PDSS; VAS-PAIN; VASF-physical; VASF-mental) status, QoL (PDQ-39SI; EUROHIS-QOL8; Figure 3), and autonomy for activities of daily living (ADLS) when compared to those without drooling (Table 1). In the PD ≤ 2 y group, drooling was associated with gait problems (FOGQ), a greater motor severity (UPDRS-III) and NMS burden (NMSS) including mood and other neuropsychiatric symptoms (BDI-II; NPI), pain (VAS-PAIN) and mental fatigue (VASF-mental), and a worse QoL (PDQ-39SI; EUROHIS-QOL8) (Table 1). Compared to patients without drooling, the frequency of major depression, freezing of gait, and falls in the subgroup of PD ≤ 2 y patients with drooling was roughly double (Table 1).

To be older (OR = 1.025; 95% CI, 1.004–1.046; *p* = 0.019), to be male (OR = 2.165; 95% CI, 1.486–3.153; *p* < 0.0001), to have a higher score on the UPDRS-III (OR = 1.018; 95% CI, 1.001–1.037; *p* = 0.047) and the NMSS (OR = 1.011; 95% CI, 1.005–1.016; *p* < 0.0001), and to have dysphagia (OR = 2.274; 95% CI, 1.476–3.505; *p* < 0.0001) were independent factors associated with drooling at the baseline (Table 2). In the PD ≤ 2 y group, a higher NMSS total score was the only independent factor associated with drooling at the baseline (OR = 1.017; 95% CI, 1.005–1.029; *p* = 0.001). Like as seen with baseline predictions, being older (OR = 1.032; 95% CI, 1.007–1.057; *p* = 0.012), being male (OR = 2.333; 95% CI, 1.540–3.536; *p* < 0.0001), having a greater NMS burden at the baseline (NMSS total score at V0; OR = 1.020; 95% CI, 1.011–1.030; *p* < 0.0001), and having a greater increase in the NMS burden from V0 to V2 (change in the NMSS total score from V0 to V2; OR = 1.012; 95% CI, 1.006–1.019; *p* < 0.0001) were identified as independent predictors of drooling after the 2-year follow-up (Table 3). When NMS burden at the baseline was considered as a categorical variable in the model, to have a very severe NMS burden at V0 (NMSS total score >70) increased the probability of drooling at V2 more than double (OR = 2.696; 95% CI, 4.248–10.729; *p* < 0.0001). Moreover, to have drooling at the baseline multiplied by 6 (OR = 6.751; 95% CI, 1.011–1.030; *p* < 0.0001), the probability of drooling at V2 after adjustment must be receiving anticholinergic drugs and the other covariates of the model. In the PD ≤ 2 y group, a higher UPDRS-III score at V0 was the only predictor of drooling at V2 identified (OR = 1.093; 95% CI, 1.025–1.166; *p* = 0.007) (Table 3). Specifically, to have at V0 a score on the UPDRS-III higher than 20 points increased the probability of drooling at V2 by 3-fold (OR = 3.671; 95% CI, 1.350–9.986; *p* = 0.011). Finally, to have a greater NMS burden at the baseline (*β* = 0.492; 95% CI, 0.052–0.089; *p* < 0.0001) and a greater increase in the NMS burden from V0 to V2 (*β* = 0.221; 95% CI, 0.020–0.048; *p* < 0.0001) were the most significant factors associated with drooling severely at V2 in the entire cohort, whereas to have at the baseline, a greater score on the UPDRS-III (*β* = 0.272; 95% CI, 0.038–0.204; *p* = 0.005) and the NMSS (*β* = 0.272; 95% CI, 0.009–0.049; *p* = 0.005) were in the PD ≤ 2 y group (Table 4). Similar results were observed when the item-19 score was excluded from the NMSS total score.

With regard to QoL, drooling was associated with a worse health-related QoL (PDQ-39SI as the dependent variable) at V0 (*β* = 0.180; 95% CI, 2.928–6.992; *p* < 0.0001) and at V2 (*β* = 0.131; 95% CI, 1.409–7.115; *p* = 0.003) and also with a worse global QoL (EUROHIS-QOL8 as dependent variable) at V0 (*β* = −0.118; 95% CI, −0.218 to −0.050; *p* = 0.002) and at V2 (*β* = −0.128; 95% CI, −0.251 to −0.047; *p* = 0.004). In the PD ≤ 2 y group, drooling was associated with a worse health-related QoL at V0 (*β* = 0.249; 95% CI, 2.855–10.362; *p* = 0.001) and at V2 (*β* = 0.306; 95% CI, 4.193–14.327; *p* < 0.0001) and with a worse global QoL at V0 (*β* = −0.238; 95% CI, −0.438 to −0.110; *p* = 0.001) as well. However, after adjustment to covariates defined in the methods, the association between drooling and both health-related and global QoL at V0 and at V2 was not significant, not even when persisting drooling or the NMSS-Drooling_V0+V1+V2_ score was considered in the model. A correlation was observed between the NMSS-Drooling_V0+V1+V2_ score and the score on both PDQ-39SI and EUROHIS-QOL8 at V2 in the entire cohort (PDQ-39SI, *r* = 0.234 (*p* < 0.0001); EUROHIS-QOL8, *r* = −0.222 (*p* < 0.0001)) and in the PD ≤ 2 y group (PDQ-39SI, *r* = 0.483 (*p* < 0.0001); EUROHIS-QOL8, *r* = −0.304 (*p* = 0.001)). QoL at V2 was worse in patients with persistent drooling in both the entire cohort (PDQ-39SI, 25.18 ± 19.14 vs. 18.4 ± 14.81 (*p* < 0.0001); EUROHIS-QOL8, 3.64 ± 0.51 vs. 3.8 ± 0.59 (*p* < 0.005)) and in the PD ≤ 2 y group (PDQ-39SI, 28.58 ± 22.71 vs. 14.03 ± 11.35 (*p* = 0.001); EUROHIS-QOL8, 3.54 ± 0.53 vs. 3.88 ± 0.57 (*p* = 0.006)). Finally, by domains, drooling was an independent factor associated with a worse “Activities of daily living” (*β* = 0.086; 95% CI, 0.654–5.925; *p* = 0.015; *R*^2^ = 0.43) and “Communication” (*β* = 0.088; 95% CI, 0.297–5.075; *p* = 0.028; *R*^2^ = 0.28) at V0 in the entire cohort.

## 4. Discussion

The present study represents one of the largest cohorts of PD patients in whom the prevalence of drooling was reported using a validated global NMS scale. We observed that drooling was common in PD patients, clearly much more frequent than in the control group, and was associated with the male gender, older age, and a greater motor and nonmotor severity. In addition, patients with drooling had a worse global and health-related QoL, although the effect of drooling on QoL was not significant after adjusting to other covariates. Importantly, we observed that drooling was also a very frequent symptom at the beginning of the disease, as seen in the very early PD patients, suggesting the clinical importance of asking for the presence of drooling at the beginning of the patient's follow-up.

About 2 out of every 3 patients from the Spanish cohort COPPADIS reported drooling over a 2-year follow-up. This cumulative prevalence is in line with the previously published data [11]. However, due to the lack of a standard definition and criteria for diagnosing drooling in PD patients, estimates of its prevalence vary considerably with a wide range from 10% to 84% [2–15]. This is partly due to different tools such as the UPDRS-II, SCOPA-AUT (Scale for Outcomes in Parkinson's disease for Autonomic Symptoms), PD-NMSQuest (Parkinson's Disease Nonmotor Symptoms Questionnaire), NMSS, or different types of screening questionnaires have been used to screen drooling in PD cohorts with different characteristics also [1, 3, 11]. Some specific scales to assess drooling have been designed, but they have been poorly used in studies with PD patients [37]. Using the NMSS-item 19 for detecting drooling like us, van Wamelen et al. [23] detected in a cohort of 728 PD patients with a mean disease duration of 5.6 years a prevalence of 37.2% at the baseline and 40.1% after a mean follow-up of 3.3 years (range 0.5–7.2 years). In many cross-sectional studies, the prevalence of drooling in PD is between 40% and 50% [2, 6–8, 11, 23, 24, 38, 39], which is in agreement with our findings. An interesting finding is that like in other studies [5, 13], drooling was not related to disease duration and in fact, the prevalence at each year (from 35% to 50%) and the cumulative prevalence after the 2-year follow-up (65%) was similar in those patients with no more than 2 years since symptom onset compared to the whole cohort. Drooling is frequent even in de novo patients. Erro et al. [27] reported in 61 de novo PD patients a frequency of drooling of 19.4% at the baseline and 15.3% after a 2-year follow-up, whereas Picillo et al. [28] found in 86 men and 48 women de novo PD patients a frequency of drooling at the baseline and after a 2-year follow-up of 23.3% and 25% and 10.4% and 4.1%, respectively. The Picillo study, in addition to ours and other studies, suggests that drooling could be more frequent in males [3, 11, 28, 40]. Although specifically well-designed studies to analyze the prevalence of drooling using specific validated scales [37, 41] in large cohorts are required, all these data suggest a recommendation to rule out drooling in PD patients at the beginning and throughout follow-up since its presence is associated with a worse QoL and it is potentially treatable. Consideration is especially valid in elderly men for which the prevalence of drooling is more frequent. Despite this, drooling is an underrecognized and undertreated symptom in PD [1]. Of note, no patient from our cohort was receiving botulinum toxin injections.

In addition to male gender, many other variables have been associated with drooling in PD patients such as dysphagia [1, 42], dysarthria [1, 43], hypomimia [14, 17], lingual bradykinesia [14, 17], cognitive status [15, 24], hallucinations [5], aging [3, 23], more advanced disease stage [16, 24], orthostatic hypotension [1], camptocormia [44], and a history of using antidepressants [6]. Drooling in PD patients can be in part due to the inability to maintain saliva in the mouth (i.e., hypomimia, abnormal flexed posture, etc.) and impairment of salivary clearance (i.e., lingual bradykinesia, oropharyngeal dysphagia, and upper esophageal dysmotility), as dysphagia is the strongest factor associated with drooling [7, 12, 23]. We identified dysphagia as a factor that doubles the probability of drooling independently of other variables, even though it was measured through patient-reported outcomes. Moreover, not only dysphagia burden but also hypomimia burden correlated with drooling burden in the entire cohort and the very early PD group as well. On the other hand, some recent studies comprehensively evaluating many features of the disease found an association between drooling and late onset of the disease, a higher LEDD, fluctuations, depression, higher motor scores, and a greater NMS burden [13–15, 23, 45]. In this Spanish cohort, we identified a greater motor severity (UPDRS-III) and a greater NMS burden (NMSS) as independent factors associated with drooling and/or also predictors of drooling after a 2-year follow-up. Specifically, a worse status in terms of motor and NMS predicted a greater drooling severity as well. This could explain why drooling was associated with a worse QoL but was not an independent predictor of it. Karakoc et al. [46] reported drooling in 65% of 63 people with PD but no independent significant correlation of drooling severity with QoL. However, as in our case, they measured the latter from the total PDQ-39 score, rather than with a tool that measures drooling impact. In contrast, when we used the PDQ-39 domains, we identified drooling as an independent factor associated with a worse autonomy for ADL (PDQ-39 domain 2) and communication (PDQ-39 domain 7). Psychosocially, PD droolers had worse QoL and had more difficulty speaking, eating, and socially interacting compared to PD nondroolers [3, 5, 11]. In addition, drooling patients affect their caregivers by increasing their burden, depression, and anxiety and reducing their QoL [47]. For all these reasons, therapeutic options should be evaluated more intensively in patients with PD and drooling [1, 11].

The present study has some important limitations. Drooling was considered based on an answer to a simple clinical question from the NMSS and not after using a specific scale [37, 41]. However, this methodology is the most frequent in most studies [2, 3, 6, 7, 11, 24, 40, 47, 48]. The sample size in the group of PD patients with no more than 2 years since the onset of the symptoms was small and clearly smaller than that of the entire cohort. In the 2-year follow-up group, there was a 30% loss in participants, although this has been observed in other cohorts, with retention rates of 71% [23], 67% [27], or 67% [28]. The logistic regression models used to identify the independent factors associated with drooling and predictors of drooling only explain 20–30% of the variance in our analysis, but it was either also low or not provided in other studies [6, 7, 13, 23]. For some variables, the information was not collected in all cases. Instead of a specific tool for assessing comorbidity, like the Charlson index or others, the total number of non-anti-Parkinsonian medications was used as a surrogate marker of comorbidity [36], and the role of possible comorbidities inducing drooling was not considered. Finally, our sample was not fully representative of the PD population due to inclusion and exclusion criteria (i.e., age limit, no dementia, no severe comorbidities, no second-line therapies, etc.) [49]. Nonetheless, the strengths of our study include a very thorough assessment, a prospective longitudinal follow-up design, and the extensive clinical and demographic information recorded. Data about drooling severity and PDQ-39 domains are novel.

In conclusion, this study observes a high prevalence of drooling in PD patients, clearly much more so than in control subjects, and that this feature is frequent even at the first stages of the disease as well. Dysphagia is associated with drooling, and a higher motor score and a greater NMS burden are predictors of drooling. PD patients with drooling have a worse QoL, and drooling is also an independent factor associated with communication problems. Thus, drooling screening and therapeutic options should be considered in clinical practice.

## Figures and Tables

**Table 1:** Disease-related characteristics, motor and nonmotor symptoms, and autonomy for activities of daily living and quality of life in patients with and without drooling at the baseline in the entire cohort (*n* = 691) and in PD ≤ 2 y (*N* = 184).

	Without drooling entire cohort (*N* = 414)	With drooling entire cohort (*N* = 277)	*p*	Without drooling PD ≤ 2 y (*N* = 120)	With drooling PD ≤ 2 y (*N* = 64)	*p*
Age	61.8 ± 9.29	63.79 ± 8.21	0.008	61.68 ± 8.54	63.39 ± 7.84	0.252
Males (%)	54.3	69	<0.0001	55	60.9	0.269
Weight (kgs)	75.37 ± 13.83	76.56 ± 13.34	0.341	75.84 ± 14.75	75.75 ± 11	0.755
Disease duration (years)	5.31 ± 4.24	5.8 ± 4.55	0.136	1.33 ± 0.73	1.22 ± 0.75	0.332
L-dopa eq. daily dose (mg)	512.73 ± 409.22	646.01 ± 410.21	<0.0001	303.51 ± 242.63	343.11 ± 256.34	0.296
Number of non antip. drugs	2.45 ± 2.43	2.79 ± 2.62	0.106	2.72 ± 2.44	2.94 ± 2.66	0.680
Motor phenotype (%)			0.899			0.995
Tremoric dominant	45.6	44.9		58.8	57.8	
PIGD	39.1	38.4		27.7	29.7	
Indeterminate	15.3	16.7		13.4	12.5	
Hoehn and Yahr-OFF	2 [1.5, 2]	2 [2, 2]	0.031	2 [1.5, 2]	2 [1.5, 2]	0.186
Stage from 3 to 5 (%)	8.6	10.5	0.257	2.9	1.7	0.526
UPDRS-III-OFF	20.97 ± 10.56	25.17 ± 11.59	<0.0001	17.56 ± 8.46	21.69 ± 9.68	0.005
Hypomimia (%)	79.8	86.9	0.011	74.6	81	0.219
UPDRS-IV	1.79 ± 2.34	2.33 ± 2.48	<0.0001	0.86 ± 1.38	1.16 ± 1.54	0.136
Motor fluctuations (%)	29.1	38.3	0.008	6.7	12.5	0.148
Dyskinesia (%)	17.5	21.2	0.137	2.6	6.6	0.190
FOGQ	3.3 ± 4.36	4.53 ± 4.78	<0.0001	1.75 ± 2.81	3.05 ± 3.66	0.031
Patients with FOG (%)	30.1	42	0.001	16.7	31.2	0.019
Patients with falls (%)	10.8	17.2	0.011	6.6	15.6	0.034
PD-CRS total score	92.52 ± 15.97	89.36 ± 15.25	0.006	92.18 ± 15.44	88.09 ± 13.73	0.077
NMSS	37.69 ± 32.09	57.28 ± 42.55	<0.0001	32.85 ± 28.08	56.4 ± 37.17	<0.0001
Dysphagia (%)	15.5	34.7	<0.0001	13.3	31.2	0.004
BDI-II	8.12 ± 7.18	9.64 ± 7.43	0.002	7.15 ± 6.98	10.81 ± 7.51	<0.0001
Major depression (%)	13.3	20.2	0.010	11.7	25	0.018
NPI	5.12 ± 6.99	7.58 ± 9.36	0.001	4.2 ± 6.52	7.34 ± 6.95	<0.0001
QUIP-RS	3.96 ± 7.63	4.97 ± 9.07	0.254	3.42 ± 7.66	2.93 ± 7.46	0.312
PDSS	116.83 ± 25.32	111.98 ± 28.8	0.027	119.45 ± 25.19	111.22 ± 32.04	0.110
VAS-PAIN	2.51 ± 2.94	2.9 ± 2.93	0.046	2.37 ± 2.9	3.18 ± 2.79	0.046
VASF − physical	2.83 ± 2.79	3.2 ± 2.68	0.038	2.55 ± 2.9	2.78 ± 2.43	0.211
VASF – mental	1.93 ± 2.5	2.47 ± 2.58	0.002	1.85 ± 2.51	2.56 ± 2.51	0.035
ADLS	89.49 ± 10.64	86.85 ± 10.15	<0.0001	92.08 ± 8.39	89.22 ± 10.12	0.053
Functional dependency (%)	8.2	10.5	0.186	4.2	7.8	0.238
PDQ-39SI	15.15 ± 12.6	20.11 ± 14.33	<0.0001	12.4 ± 11.33	19.01 ± 13.91	<0.0001
EUROHIS-QOL8	3.83 ± 0.54	2.71 ± 0.56	0.005	3.91 ± 0.56	3.64 ± 0.48	0.001

The results represent percentages, mean ± SD, or median (p25, p75). The chi-squared and Mann-Whitney-Wilcoxon tests were applied for comparisons between patients with and without drooling at the baseline. Data about H&Y and UPDRS-III are during the OFF state (first thing in the morning without taking medication in the previous 12 hours). ADLS: Schwab and England Activities of Daily Living Scale); antip.: antiparkinsonian; BDI: Beck Depression Inventory-II; NMSS: Nonmotor Symptoms Scale; NPI: Neuropsychiatric Inventory; PD: Parkinson's disease; PD ≤ 2 y: PD with ≤2 years from symptom onset; PD-CRS: Parkinson's Disease Cognitive Rating Scale; PDSS: Parkinson's Disease Sleep Scale; PIGD: Postural Instability Gait Difficulty; QUIP-RS: Questionnaire for Impulsive-Compulsive Disorders in Parkinson's Disease-Rating Scale; UPDRS: Unified Parkinson's Disease Rating Scale; VAFS: Visual Analog Fatigue Scale; VAS-Pain: Visual Analog Scale-Pain.

**Table 2:** Factors associated with drooling at the baseline in the entire cohort (*n* = 691) and in the PD ≤ 2 y group (*N* = 184).

	OR^a^	OR^b^	95% CI^a^	95% CI^b^	*p* ^a^	*p* ^b^
Entire cohort						
Age	1.026	1.025	1.008–1.044	1.004–1.046	0.004	0.019
Male	1.806	2.165	1.308–2.493	1.486–3.153	<0.0001	<0.0001
LEDD	1.001	1.000	1.001–1.002	1.000–1.001	<0.0001	0.172
UPDRS-III	1.035	1.018	1.020–1.050	1.001–1.037	<0.0001	0.047
NMSS	1.015	1.011	1.010–1.019	1.005–1.016	<0.0001	<0.0001
Dysphagia	2.901	2.274	2.016–4.173	1.476–3.505	<0.0001	<0.0001
PD ≤ 2 y group						
UPDRS-III	1.052	1.034	1.014–1.090	0.994–1.076	0.006	0.096
NMSS	1.022	1.017	1.012–1.033	1.005–1.029	<0.0001	0.004
Dysphagia	2.995	2.002	1.401–6.229	0.858–4.672	0.004	0.108

Dependent variable: drooling at V0 (NMSS-item 19 ≥ 1). OR (odds ratio) and 95% ICare shown. ^a^univariate analysis; ^b^multivariate analysis; entire cohort, *R*^2^ = 0.19; Hosmer and Lemeshow test, *p* = 0.226; PD ≤ 2 y, *R*^2^ = 0.19; Hosmer and Lemeshow test, *p* = 0.774. LEED: levodopa equivalent daily dose (mg/day); NMSS: Nonmotor Symptoms Scale; PD ≤ 2 y: PD with ≤2 years from symptom onset; UPDRS: Unified Parkinson's Disease Rating Scale.

**Table 3:** Predictors of drooling after the 2-year follow-up in the entire cohort (*N* = 481) and in the PD ≤ 2 y group (*N* = 127).

	OR^a^	OR^b^	95% CI^a^	95% CI^b^	*p* ^a^	*p* ^b^
Entire cohort						
Age	1.033	1.032	1.011–1.056	1.007–1.057	0.003	0.012
Male	2.023	2.333	1.396–2.932	1.540–3.536	<0.0001	<0.0001
UPDRS-III at V0	1.028	1.016	1.010–1.047	0.995–1.038	0.002	0.097
NMSS at V0	1.010	1.020	1.005–1.016	1.011–1.030	<0.0001	<0.0001
PDQ-39SI at V0	1.016	0.978	1.002–1.031	0.955–1.002	0.024	0.069
Change from V0 to V2 in NMSS	1.006	1.012	1.001–1.011	1.006–1.019	0.042	<0.0001
PD ≤ 2 y group						
Age	1.037	1.037	0.994–1.082	0.984–1.092	0.096	0.098
Male	1.707	2.064	0.845–3.450	0.886–4.810	0.136	0.093
UPDRS-III at V0	1.121	1.093	1.056–1.191	1.025–1.166	<0.0001	0.007
NMSS at V0	1.019	1.013	1.007–1.032	0.998–1.032	0.128	0.082

Dependent variable: drooling at V2 (NMSS-item 19 ≥ 1). OR (odds ratio) and 95% IC are shown. ^a^univariate analysis; ^b^multivariate analysis; entire cohort, *R*^2^ = 0.33; Hosmer and Lemeshow test, *p* = 0.163; PD ≤ 2 y, *R*^2^ = 26; Hosmer and Lemeshow test, *p* = 0.788. LEED: levodopa equivalent daily dose (mg/day); NMSS: Nonmotor Symptoms Scale; PD ≤ 2 y: PD with ≤2 years from symptom onset; PDQ-39SI: 39-item Parkinson's disease Questionnaire Summary Index; UPDRS: Unified Parkinson's Disease Rating Scale.

**Table 4:** Factors associated with drooling severity after the 2-year follow-up in the entire cohort (*N* = 481) and in the PD ≤ 2 y group (*N* = 127).

	*β* ^a^	*β* ^b^	95% CI^a^	95% CI^b^	*p* ^a^	*p* ^b^
Entire cohort						
Age	0.114	0.083	0.015–0.127	−0.001–0.109	0.013	0.052
Male	0.112	0.136	0.252–2.199	0.568–2.445	0.014	0.002
UPDRS-III at V0	0.207	0.087	0.060–1.054	−0.003–0.093	<0.0001	0.068
NMSS at V0	0.359	0.492	0.039–0.063	0.052–0.089	<0.0001	<0.0001
PDQ-39SI at V0	0.240	−0.110	0.063–1.135	−0.098–0.007	<0.0001	0.087
Change from V0 to V2 in NMSS	0.073	0.221	−0.003–0.125	0.020–0.048	0.110	<0.0001
PD ≤ 2 y group						
UPDRS-III at V0	1.121	0.272	1.056–1.191	0.038–0.204	<0.0001	0.005
NMSS at V0	1.019	0.272	1.007–1.032	0.009–0.049	<0.0001	0.005

Dependent variable: drooling_V0+V1+V2_ score. *β* standardized coefficient and 95% IC are shown. ^a^univariate analysis; ^b^multivariate analysis; entire cohort, *R*^2^ = 0.21; Durbin–Watson test = 1.92; PD ≤ 2 y, *R*^2^ = 22; Durbin–Watson test = 1.94. NMSS: Nonmotor Symptoms Scale; PD ≤ 2 y: PD with ≤2 years from symptom onset; PDQ-39SI: 39-item Parkinson's disease Questionnaire Summary Index; UPDRS: Unified Parkinson's Disease Rating Scale.

**Table 5:** COPPADIS study group.

Name (last name, first name)	Location	Role	Contribution
Astrid Adarmes, Daniela	Hospital Universitario Virgen del Rocío, Sevilla, Spain	Site investigator	Evaluation of participants and/or data management
Almeria, Marta	Hospital Universitari Mutua de Terrassa, Terrassa, Barcelona, Spain	Site investigator	Neuropsychologist; evaluation of participants
Alonso Losada, Maria Gema	Hospital Álvaro Cunqueiro, Complejo Hospitalario Universitario de Vigo (CHUVI), Vigo, Spain	Site investigator/PI	Coordination at the center evaluation of participants and/or data management
Alonso Cánovas, Araceli	Hospital Universitario Ramón y Cajal, Madrid, Spain	Site investigator	Evaluation of participants and/or data management
Alonso Frech, Fernando	Hospital Universitario Clínico San Carlos, Madrid, Spain	Site investigator	Evaluation of participants and/or data management
Alonso Redondo, Ruben	Hospital Universitario Lucus Augusti (HULA), Lugo, Spain	Site investigator/PI	Coordination at the center evaluation of participants and/or data management
Aneiros Díaz, Ángel	Complejo Hospitalario Universitario de Ferrol (CHUF), Ferrol, A Coruña, Spain	Site investigator/PI	Coordination at the center evaluation of participants and/or data management
Álvarez, Ignacio	Hospital Universitari Mutua de Terrassa, Terrassa, Barcelona, Spain	Site investigator	Evaluation of participants and/or data management
Álvarez Sauco, María	Hospital General Universitario de Elche, Elche, Spain	Site investigator/PI	Coordination at the center evaluation of participants and/or data management
Arnáiz, Sandra	Complejo Asistencial Universitario de Burgos, Burgos, Spain	Site investigator	Evaluation of participants and/or data management
Arribas, Sonia	Hospital Universitari Mutua de Terrassa, Terrassa, Barcelona, Spain	Site investigator	Neuropsychologist; evaluation of participants
Ascunce Vidondo, Arancha	Complejo Hospitalario de Navarra, Pamplona, Spain	Site investigator	Evaluation of participants and/or data management
Aguilar, Miquel	Hospital Universitari Mutua de Terrassa, Terrassa, Barcelona, Spain	Site investigator	Evaluation of participants and/or data management
Ávila Rivera, Maria Asunción	Consorci Sanitari Integral, Hospital General de L´Hospitalet, L´Hospitalet de Llobregat, Barcelona, Spain	Site investigator/PI	Coordination at the center evaluation of participants and/or data management
Bernardo Lambrich, Noemí	Hospital de Tortosa Verge de la Cinta (HTVC), Tortosa, Tarragona, Spain	Site investigator	Evaluation of participants and/or data management
Bejr-Kasem, Helena	Hospital de Sant Pau, Barcelona, Spain	Site investigator	Evaluation of participants and/or data management
Blázquez Estrada, Marta	Hospital Universitario Central de Asturias, Oviedo, Spain	Site investigator	Evaluation of participants and/or data management
Botí González, Maria Ángeles	Hospital Universitari Mutua de Terrassa, Terrassa, Barcelona, Spain	Site investigator	Neuropsychologist; evaluation of participants
Borrué, Carmen	Hospital Infanta Sofía, Madrid, Spain	Site investigator/PI	Coordination at the center evaluation of participants and/or data management
Buongiorno, Maria Teresa	Hospital Universitari Mutua de Terrassa, Terrassa, Barcelona, Spain	Site investigator	Nurse study coordinator
Cabello González, Carolina	Complejo Hospitalario de Navarra, Pamplona, Spain	Site investigator	Scheduling of evaluations
Cabo López, Iria	Complejo Hospitalario Universitario de Pontevedra (CHOP), Pontevedra, Spain	Site investigator/PI	Coordination at the center evaluation of participants and/or data management
Caballol, Nuria	Consorci Sanitari Integral, Hospital Moisés Broggi, Sant Joan Despí, Barcelona, Spain	Site investigator/PI	Coordination at the center evaluation of participants and/or data management
Cámara Lorenzo, Ana	Hospital Clínic de Barcelona, Barcelona, Spain	Site investigator	Nurse study coordinator
Canfield Medina, Héctor	Complejo Hospitalario Universitario de Ferrol (CHUF), Ferrol, A Coruña, Spain	Site investigator	Evaluation of participants and/or data management
Carrillo, Fátima	Hospital Universitario Virgen del Rocío, Sevilla, Spain	Site investigator	Evaluation of participants and/or data management
Carrillo Padilla, Francisco José	Hospital Universitario de Canarias, San Cristóbal de la Laguna, Santa Cruz de Tenerife, Spain	Site investigator/PI	Coordination at the center evaluation of participants and/or data management
Casas, Elena	Complejo Asistencial Universitario de Burgos, Burgos, Spain	Site investigator	Evaluation of participants and/or data management
Catalán, Maria José	Hospital Universitario Clínico San Carlos, Madrid, Spain	Site investigator/PI	Coordination at the center evaluation of participants and/or data management
Clavero, Pedro	Complejo Hospitalario de Navarra, Pamplona, Spain	Site investigator	Evaluation of participants and/or data management
Cortina Fernández, A	Complejo Hospitalario Universitario de Ferrol (CHUF), Ferrol, A Coruña, Spain	Site investigator	Coordination of blood extractions
Cosgaya, Marina	Hospital Clínic de Barcelona, Barcelona, Spain	Site investigator	Evaluation of participants and/or data management
Cots Foraster, Ana	Institut d' Assistència Sanitària (IAS) - Instituí Cátala de la Salud. Girona, Spain	Site investigator	Evaluation of participants and/or data management
Crespo Cuevas, Ane	Hospital del Mar, Barcelona, Spain	Site investigator	Evaluation of participants and/or data management
Cubo, Esther	Complejo Asistencial Universitario de Burgos, Burgos, Spain	Site investigator/PI	Coordination at the center evaluation of participants and/or data management
De Deus Fonticoba, Teresa	Complejo Hospitalario Universitario de Ferrol (CHUF), Ferrol, A Coruña, Spain	Site investigator	Nurse study coordinator evaluation of participants and/or data management
De Fábregues-Boixar, Oriol	Hospital Universitario Vall d´Hebron, Barcelona, Spain	Site investigator/PI	Coordination at the center evaluation of participants and/or data management
Díez Fairen, M	Hospital Universitari Mutua de Terrassa, Terrassa, Barcelona, Spain	Site investigator	Evaluation of participants and/or data management
Dotor García-Soto, Julio	Hospital Universitario Virgen Macarena, Sevilla, Spain	Site investigator/PI	Evaluation of participants and/or data management
Erro, Elena	Complejo Hospitalario de Navarra, Pamplona, Spain	Site investigator	Evaluation of participants and/or data management
Escalante, Sonia	Hospital de Tortosa Verge de la Cinta (HTVC), Tortosa, Tarragona, Spain	Site investigator/PI	Coordination at the center evaluation of participants and/or data management
Estelrich Peyret, Elena	Institut d' Assistència Sanitària (IAS) - Instituí Cátala de la Salud. Girona, Spain	Site investigator	Evaluation of participants and/or data management
Fernández Guillán, Noelia	Complejo Hospitalario Universitario de Ferrol (CHUF), Ferrol, A Coruña, Spain	Site investigator	Neuroimaging studies
Gámez, Pedro	Complejo Asistencial Universitario de Burgos, Burgos, Spain	Site investigator	Evaluation of participants and/or data management
Gallego, Mercedes	Hospital La Princesa, Madrid, Spain	Site investigator	Evaluation of participants and/or data management
García Caldentey, Juan	Centro Neurológico Oms 42, Palma de Mallorca, Spain	Site investigator/PI	Coordination at the center evaluation of participants and/or data management
García Campos, Cristina	Hospital Universitario Virgen Macarena, Sevilla, Spain	Site investigator	Evaluation of participants and/or data management
García Díez, Cristina	Complejo Hospitalario Universitario de Pontevedra (CHOP), Pontevedra, Spain	Site investigator (from MAY/22)	Neuropsychologist; evaluation of participants
García Moreno, José Manuel	Hospital Universitario Virgen Macarena, Sevilla, Spain	Site investigator/PI (until MAR/21)	Coordination at the center evaluation of participants and/or data management
Gastón, Itziar	Complejo Hospitalario de Navarra, Pamplona, Spain	Site investigator/PI	Coordination at the center evaluation of participants and/or data management
Gómez Garre, María del Pilar	Hospital Universitario Virgen del Rocío, Sevilla, Spain	Site investigator	Genetic studies coordination
Gómez Mayordomo, Víctor	Hospital Clínico San Carlos, Madrid, Spain	Site investigator	Evaluation of participants and/or data management
González Aloy, Javier	Institut d' Assistència Sanitària (IAS) - Instituí Cátala de la Salud. Girona, Spain	Site investigator	Evaluation of participants and/or data management
González Aramburu, Isabel	Hospital Universitario Marqués de Valdecilla, Santander, Spain	Site investigator	Evaluation of participants and/or data management
González Ardura, Jessica	Hospital Universitario Lucus Augusti (HULA), Lugo, Spain	Site investigator/PI (until FEB/21)	Evaluation of participants and/or data management
González García, Beatriz	Hospital La Princesa, Madrid, Spain	Site investigator	Nurse study coordinator
González Palmás, Maria Josefa	Complejo Hospitalario Universitario de Pontevedra (CHOP), Pontevedra, Spain	Site investigator	Evaluation of participants and/or data management
González Toledo, Gabriel Ricardo	Hospital Universitario de Canarias, San Cristóbal de la Laguna, Santa Cruz de Tenerife, Spain	Site investigator	Evaluation of participants and/or data management
Golpe Díaz, Ana	Complejo Hospitalario Universitario de Ferrol (CHUF), Ferrol, A Coruña, Spain	Site investigator	Laboratory analysis coordination
Grau Solá, Mireia	Consorci Sanitari Integral, Hospital Moisés Broggi, Sant Joan Despí, Barcelona, Spain	Site investigator	Evaluation of participants and/or data management
Guardia, Gema	Hospital Universitari Mutua de Terrassa, Terrassa, Barcelona, Spain	Site investigator	Evaluation of participants and/or data management
Hernández Vara, Jorge	Hospital Universitario Vall d´Hebron, Barcelona, Spain	Site investigator/PI	Coordination at the center evaluation of participants and/or data management
Horta Barba, Andrea	Hospital de Sant Pau, Barcelona, Spain	Site investigator	Neuropsychologist; evaluation of participants
Idoate Calderón, Daniel	Complejo Hospitalario Universitario de Pontevedra (CHOP), Pontevedra, Spain	Site investigator (until MAY/22)	Neuropsychologist; evaluation of participants
Infante, Jon	Hospital Universitario Marqués de Valdecilla, Santander, Spain	Site investigator/PI	Coordination at the center evaluation of participants and/or data management
Jesús, Silvia	Hospital Universitario Virgen del Rocío, Sevilla, Spain	Site investigator	Evaluation of participants and/or data management
Kulisevsky, Jaime	Hospital de Sant Pau, Barcelona, Spain	Site investigator/PI	Coordination at the center evaluation of participants and/or data management
Kurtis, Mónica	Hospital Ruber Internacional, Madrid, Spain	Site investigator/PI	Coordination at the center evaluation of participants and/or data management
Labandeira, Carmen	Hospital Álvaro Cunqueiro, Complejo Hospitalario Universitario de Vigo (CHUVI), Vigo, Spain	Site investigator	Evaluation of participants and/or data management
Labrador Espinosa, Miguel Ángel	Hospital Universitario Virgen del Rocío, Sevilla, Spain	Site investigator	Neuroimaging data analysis
Lacruz, Francisco	Complejo Hospitalario de Navarra, Pamplona, Spain	Site investigator	Evaluation of participants and/or data management
Lage Castro, Melva	Complejo Hospitalario Universitario de Pontevedra (CHOP), Pontevedra, Spain	Site investigator	Evaluation of participants and/or data management
Lastres Gómez, Sonia	Complejo Hospitalario Universitario de Pontevedra (CHOP), Pontevedra, Spain	Site investigator	Neuropsychologist; evaluation of participants
Legarda, Inés	Hospital Universitario Son Espases, Palma de Mallorca, Spain	Site investigator/PI	Coordination at the center evaluation of participants and/or data management
López Ariztegui, Nuria	Complejo Hospitalario de Toledo, Toledo, Spain	Site investigator/PI	Evaluation of participants and/or data management
López Díaz, Luis Manuel	Hospital Da Costa de Burela, Lugo, Spain	Site investigator	Evaluation of participants and/or data management
López Domínguez, Daniel	Institut d' Assistència Sanitària (IAS) - Instituí Cátala de la Salud. Girona, Spain	Site investigator	Evaluation of participants and/or data management
López Manzanares, Lydia	Hospital La Princesa, Madrid, Spain	Site investigator/PI	Coordination at the center evaluation of participants and/or data management
López Seoane, Balbino	Complejo Hospitalario Universitario de Ferrol (CHUF), Ferrol, A Coruña, Spain	Site investigator	Neuroimaging studies
Lucas del Pozo, Sara	Hospital Universitario Vall d´Hebron, Barcelona, Spain	Site investigator	Evaluation of participants and/or data management
Macías, Yolanda	Fundación Hospital de Alcorcón, Madrid, Spain	Site investigator	Evaluation of participants and/or data management
Mata, Marina	Hospital Infanta Sofía, Madrid, Spain	Site investigator	Evaluation of participants and/or data management
Martí Andres, Gloria	Hospital Universitario Vall d´Hebron, Barcelona, Spain	Site investigator	Evaluation of participants and/or data management
Martí, Maria José	Hospital Clínic de Barcelona, Barcelona, Spain	Site investigator/PI	Coordination at the center evaluation of participants and/or data management
Martínez Castrillo, Juan Carlos	Hospital Universitario Ramón y Cajal, Madrid, Spain	Site investigator/PI	Coordination at the center evaluation of participants and/or data management
Martinez-Martin, Pablo	Centro Nacional de Epidemiología y CIBERNED, Instituto de Salud Carlos III. Madrid	Collaborator in statistical and methods analysis	Methods and statistical reviewer
McAfee, Darrian	University of Pennsylvania, Philadelphia	Collaborator in English style	English style reviewer
Meitín, Maria Teresa	Hospital Da Costa de Burela, Lugo, Spain	Site investigator	Evaluation of participants and/or data management
Menéndez González, Manuel	Hospital Universitario Central de Asturias, Oviedo, Spain	Site investigator/PI	Coordination at the center evaluation of participants and/or data management
Méndez del Barrio, Carlota	Hospital Universitario Virgen del Rocío, Sevilla, Spain	Site investigator	Evaluation of participants and/or data management
Mendoza Plasencia, Zebenzui	Hospital Universitario de Canarias, San Cristóbal de la Laguna, Santa Cruz de Tenerife, Spain	Site investigator	Evaluation of participants and/or data management
Mir, Pablo	Hospital Universitario Virgen del Rocío, Sevilla, Spain	Site investigator/PI	Coordination at the center evaluation of participants and/or data management
Miranda Santiago, Javier	Complejo Asistencial Universitario de Burgos, Burgos, Spain	Site investigator	Evaluation of participants and/or data management
Morales Casado, Maria Isabel	Complejo Hospitalario de Toledo, Toledo, Spain	Site investigator	Evaluation of participants and/or data management
Moreno Diéguez, Antonio	Complejo Hospitalario Universitario de Ferrol (CHUF), Ferrol, A Coruña, Spain	Site investigator	Neuroimaging studies
Nogueira, Víctor	Hospital Da Costa de Burela, Lugo, Spain	Site investigator/PI	Coordination at the center evaluation of participants and/or data management
Novo Amado, Alba	Complejo Hospitalario Universitario de Ferrol (CHUF), Ferrol, A Coruña, Spain	Site investigator	Neuroimaging studies
Novo Ponte, Sabela	Hospital Universitario Puerta de Hierro, Madrid, Spain	Site investigator	Evaluation of participants and/or data management
Ordás, Carlos	Hospital Rey Juan Carlos, Madrid, Spain, Madrid, Spain	Site Investigator	Evaluation of participants and/or data management
Pagonabarraga, Javier	Hospital de Sant Pau, Barcelona, Spain	Site investigator	Evaluation of participants and/or data management
Pareés, Isabel	Hospital Ruber Internacional, Madrid, Spain	Site investigator	Evaluation of participants and/or data management
Pascual-Sedano, Berta	Hospital de Sant Pau, Barcelona, Spain	Site Investigator	Evaluation of participants and/or data management
Pastor, Pau	Hospital Universitari Mutua de Terrassa, Terrassa, Barcelona, Spain	Site investigator	Evaluation of participants and/or data management
Pérez Fuertes, Aída	Complejo Hospitalario Universitario de Ferrol (CHUF), Ferrol, A Coruña, Spain	Site investigator	Blood analysis
Pérez Noguera, Rafael	Hospital Universitario Virgen Macarena, Sevilla, Spain	Site investigator	Evaluation of participants and/or data management
Planas-Ballvé, Ana	Consorci Sanitari Integral, Hospital Moisés Broggi, Sant Joan Despí, Barcelona, Spain	Site investigator	Evaluation of participants and/or data management
Planellas, Lluís	Hospital Clínic de Barcelona, Barcelona, Spain	Site investigator (until DEC/19)	Evaluation of participants and/or data management
Prats, Marian Ángeles	Institut d' Assistència Sanitària (IAS) - Instituí Cátala de la Salud. Girona, Spain	Site investigator	Evaluation of participants and/or data management
Prieto Jurczynska, Cristina	Hospital Rey Juan Carlos, Madrid, Spain, Madrid, Spain	Site investigator/PI	Coordination at the center evaluation of participants and/or data management
Puente, Víctor	Hospital del Mar, Barcelona, Spain	Site investigator/PI	Coordination at the center evaluation of participants and/or data management
Pueyo Morlans, Mercedes	Hospital Universitario de Canarias, San Cristóbal de la Laguna, Santa Cruz de Tenerife, Spain	Site investigator	Evaluation of participants and/or data management
Puig Daví, Arnau	Hospital de Sant Pau, Barcelona, Spain	Site investigator	Evaluation of participants and/or data management
Redondo, Nuria	Hospital La Princesa, Madrid, Spain	Site investigator	Evaluation of participants and/or data management
Rodríguez Méndez, Luisa	Complejo Hospitalario Universitario de Ferrol (CHUF), Ferrol, A Coruña, Spain	Site investigator	Blood analysis
Rodríguez Pérez, Amparo Belén	Hospital General Universitario de Elche, Elche, Spain	Site investigator	Evaluation of participants and/or data management
Roldán, Florinda	Hospital Universitario Virgen del Rocío, Sevilla, Spain	Site investigator	Neuroimaging studies
Ruíz de Arcos, María	Hospital Universitario Virgen Macarena, Sevilla, Spain	Site investigator	Evaluation of participants and/or data management
Ruíz Martínez, Javier	Hospital Universitario Donostia, San Sebastián, Spain	Site investigator	Evaluation of participants and/or data management
Sánchez Alonso, Pilar	Hospital Universitario Puerta de Hierro, Madrid, Spain	Site investigator	Evaluation of participants and/or data management
Sánchez-Carpintero, Macarena	Complejo Hospitalario Universitario de Ferrol (CHUF), Ferrol, A Coruña, Spain	Site investigator	Neuroimaging studies
Sánchez Díez, Gema	Hospital Universitario Ramón y Cajal, Madrid, Spain	Site investigator	Evaluation of participants and/or data management
Sánchez Rodríguez, Antonio	Hospital Universitario Marqués de Valdecilla, Santander, Spain	Site investigator	Evaluation of participants and/or data management
Santacruz, Pilar	Hospital Clínic de Barcelona, Barcelona, Spain	Site investigator	Evaluation of participants and/or data management
Santos García, Diego	CHUAC, Complejo Hospitalario Universitario de A Coruña	Coordinator of the project	Coordination of the COPPADIS-2015
Segundo Rodríguez, José Clemente	Complejo Hospitalario de Toledo, Toledo, Spain	Site investigator	Evaluation of participants and/or data management
Seijo, Manuel	Complejo Hospitalario Universitario de Pontevedra (CHOP), Pontevedra, Spain	Site investigator/PI	Coordination at the center evaluation of participants and/or data management
Sierra, María	Hospital Universitario Marqués de Valdecilla, Santander, Spain	Site investigator	Evaluation of participants and/or data management
Solano, Berta	Institut d' Assistència Sanitària (IAS) - Instituí Cátala de la Salud. Girona, Spain	Site investigator/PI	Coordination at the center evaluation of participants and/or data management
Suárez Castro, Ester	Complejo Hospitalario Universitario de Ferrol (CHUF), Ferrol, A Coruña, Spain	Site investigator	Evaluation of participants and/or data management
Tartari, Juan Pablo	Hospital Universitari Mutua de Terrassa, Terrassa, Barcelona, Spain	Site investigator	Evaluation of participants and/or data management
Valero, Caridad	Hospital Arnau de Vilanova, Valencia, Spain	Site investigator	Evaluation of participants and/or data management
Vargas, Laura	Hospital Universitario Virgen del Rocío, Sevilla, Spain	Site investigator	Evaluation of participants and/or data management
Vela, Lydia	Fundación Hospital de Alcorcón, Madrid, Spain	Site investigator/PI	Coordination at the center evaluation of participants and/or data management
Villanueva, Clara	Hospital Universitario Clínico San Carlos, Madrid, Spain	Site investigator	Evaluation of participants and/or data management
Vives, Bárbara	Hospital Universitario Son Espases, Palma de Mallorca, Spain	Site investigator	Evaluation of participants and/or data management

## Data Availability

The protocol and the statistical analysis plan are available on request. Deidentified participants data are not available for legal and ethical reasons.

## Appendix

### A. Coppadis Study Group

Adarmes AD, Almeria M, Alonso Losada MG, Alonso Cánovas A, Alonso Frech F, Alonso Redondo R, Álvarez The authors, Álvarez Sauco M, Aneiros Díaz A, Arnáiz S, Arribas S, Ascunce Vidondo A, Aguilar M, Ávila MA, Bernardo Lambrich N, Bejr-Kasem H, Blázquez Estrada M, Botí M, Borrue C, Buongiorno MT, Cabello González C, Cabo López The authors, Caballol N, Cámara Lorenzo A, Canfield Medina H, Carrillo F, Carrillo Padilla FJ, Casas E, Catalán MJ, Clavero P, Cortina Fernández A, Cosgaya M, Cots Foraster A, Crespo Cuevas A, Cubo E, de Deus Fonticoba T, de Fábregues-Boixar O, Díez-Fairen M, Dotor García-Soto J, Erro E, Escalante S, Estelrich Peyret E, Fernández Guillán N, Gámez P, Gallego M, García Caldentey J, García Campos C, García Díez C, García Moreno JM, Gastón The authors, Gómez Garre MP, Gómez Mayordomo V, González Aloy J, González-Aramburu The authors, González Ardura J, González García B, González Palmás MJ, González Toledo GR, Golpe Díaz A, Grau Solá M, Guardia G, Hernández Vara J, Horta-Barba A, Idoate Calderón D, Infante J, Jesús S, Kulisevsky J, Kurtis M, Labandeira C, Labrador MA, Lacruz F, Lage Castro M, Lastres Gómez S, Legarda The authors, López Ariztegui N, López Díaz LM, López Domínguez D, López Manzanares L, López Seoane B, Lucas del Pozo S, Macías Y, Mata M, Martí Andres G, Martí MJ, Martínez Castrillo JC, Martinez-Martin P, McAfee D, Meitín MT, Mendoza Plasencia Z, Menéndez González M, Méndez del Barrio C, Mir P, Miranda Santiago J, Morales Casado MI, Moreno Diéguez A, Nogueira V, Novo Amado A, Novo Ponte S, Ordás C, Pagonabarraga J, Pareés The authors, Pascual-Sedano B, Pastor P, Pérez Fuertes A, Pérez Noguera R, Planas-Ballvé A, Planellas L, Prats MA, Prieto Jurczynska C, Puente V, Pueyo Morlans M, Puig Daví A, Redondo Rafales N, Rodríguez Méndez L, Rodríguez Pérez AB, Roldán F, Ruíz De Arcos M, Ruíz Martínez J, Sánchez Alonso P, Sánchez-Carpintero M, Sánchez Díez G, Sánchez Rodríguez A, Santacruz P, Santos García D, Segundo Rodríguez JC, Seijo M, Sierra Peña M, Solano Vila B, Suárez Castro E, Tartari JP, Valero C, Vargas L, Vela L, Villanueva C, Vives B (Table 5).

## Abbreviations

ADLS: Schwab & England activities of daily living scale
BDI-II: Beck depression inventory-II
EUROHIS-QOL8: EUROHIS-QOL 8-item index
FOG-Q: Freezing of gait questionnaire
LEDD: Levodopa equivalent daily dose
NMS: Non-motor symptoms
NMSS: Non-motor symptoms scale
NPI: Neuropsychiatric inventory
PD: Parkinson's disease
PD-CRS: Parkinson's disease cognitive rating scale
PDQ-39SI: 39-item Parkinson's disease quality of life questionnaire summary index
PDSS: Parkinson's disease sleep scale
QoL: Quality of life
QUIP-RS: Questionnaire for impulsive-compulsive disorders in Parkinson's disease-rating scale
UPDRS: Unified Parkinson's disease rating scale
VAFS: Visual analog fatigue scale
VAS-Pain: Visual analog scale-pain.
